# Do Foliar Endophytes Matter in Litter Decomposition?

**DOI:** 10.3390/microorganisms8030446

**Published:** 2020-03-21

**Authors:** Emily R. Wolfe, Daniel J. Ballhorn

**Affiliations:** Department of Biology, Portland State University, Portland, OR 97201, USA; ballhorn@pdx.edu

**Keywords:** plant–microbe interactions, ecosystem processes, microbe–microbe interactions, fungi, bacteria

## Abstract

Litter decomposition rates are affected by a variety of abiotic and biotic factors, including the presence of fungal endophytes in host plant tissues. This review broadly analyzes the findings of 67 studies on the roles of foliar endophytes in litter decomposition, and their effects on decomposition rates. From 29 studies and 1 review, we compiled a comprehensive table of 710 leaf-associated fungal taxa, including the type of tissue these taxa were associated with and isolated from, whether they were reported as endo- or epiphytic, and whether they had reported saprophytic abilities. Aquatic (i.e., in-stream) decomposition studies of endophyte-affected litter were significantly under-represented in the search results (*p* < 0.0001). Indicator species analyses revealed that different groups of fungal endophytes were significantly associated with cool or tropical climates, as well as specific plant host genera (*p* < 0.05). Finally, we argue that host plant and endophyte interactions can significantly influence litter decomposition rates and should be considered when interpreting results from both terrestrial and in-stream litter decomposition experiments.

## 1. Introduction

Litter decomposition is an essential ecosystem process that significantly contributes to the global carbon cycle. Numerous studies have identified several overarching controls on litter decomposition rates, including temperature, dissolved oxygen in aqueous environments, soil moisture, seasonality, quantity of litter pulses, and litter chemistry or quality [1,2,3]. However, litter chemistry is unique among these factors in that it may be mediated by both abiotic—e.g., drought and nutrient availability [4,5]—as well as biotic factors, such as herbivores [6], microbial symbionts [7], and pathogens [8].

Changes in litter chemistry primarily affect decomposition rates by influencing interactions with macroinvertebrate and microbial decomposer communities, which mechanically process litter and break down recalcitrant compounds, respectively. Chomel et al. [9] reviewed the generally recalcitrant properties of alkaloids, phenolic compounds, and terpenes in litter decomposition, but only briefly covered the roles of endophytes—ubiquitous microbes that mostly live asymptomatically within host plant tissues—in regulating the production of these secondary metabolites in host plants. Both endophytic fungi and bacteria were isolated from healthy plant tissues, including stems, leaves, and roots [10]; their *in planta* functions are mostly unknown. Fungal endophytes were most extensively studied in agriculturally significant grass hosts (i.e., Class 1, or clavicipitaceous endophytes; e.g., [11]), where these systemic endophytes were shown to produce toxic alkaloids (e.g., [8,12]), and even alter plant community assembly [13,14]. Furthermore, these systemic endophytes were reported to cause slower rates of litter decomposition in terrestrial systems [15,16]. However, few studies examined the role of foliar endophytes in the decomposition of litter from non-grass hosts (i.e., Class 2 or 3, or non-clavicipitaceous endophytes), and even fewer studies focused on the effects of endophytes on leaf litter decomposition in aquatic systems. Although ubiquitous within plant tissues, endophyte communities can vary spatially and temporally within host plants [17] and represent an important bridge between host plant characteristics that influence decomposition and the decomposer community.

Here, we aimed at distilling the available literature on the effects of foliar endophytes on leaf litter decomposition into an up-to-date review. We used 67 published studies and compiled a report analyzing the contributions of foliar endophytes to litter decomposition rates. We further collected information about the reported taxa of both endophytic and epiphytic fungi recovered from leaf tissue and constructed a comprehensive table for reference.

## 2. Materials and Methods

**Figure 1:**
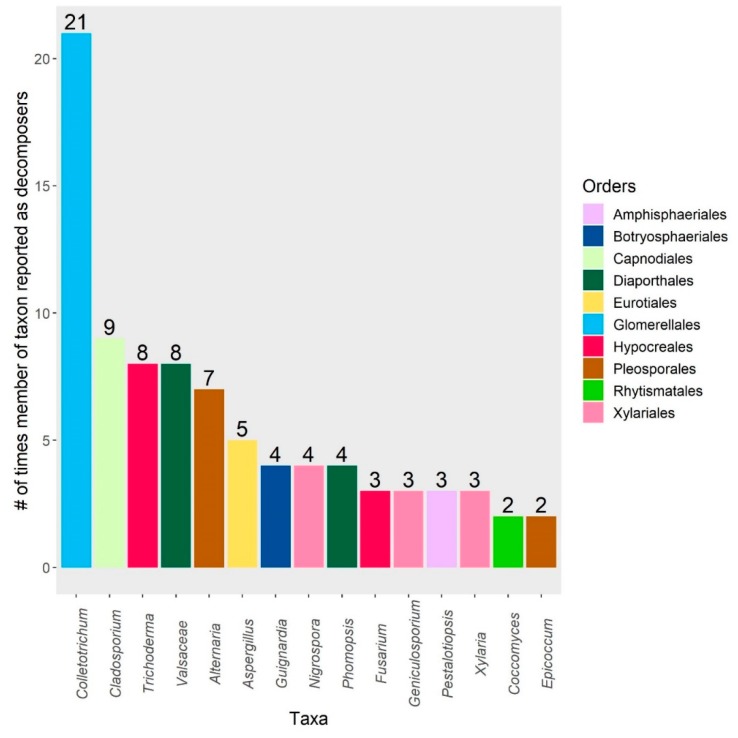
The 15 most frequently reported taxa that have measurable decomposition or saprophytic ability (e.g., cellulase secretion, etc.). Bars represent the number of times members of a taxon were reported. Taxa identified as “undetermined” or “Fungal sp.” are excluded.

**Figure 2:**
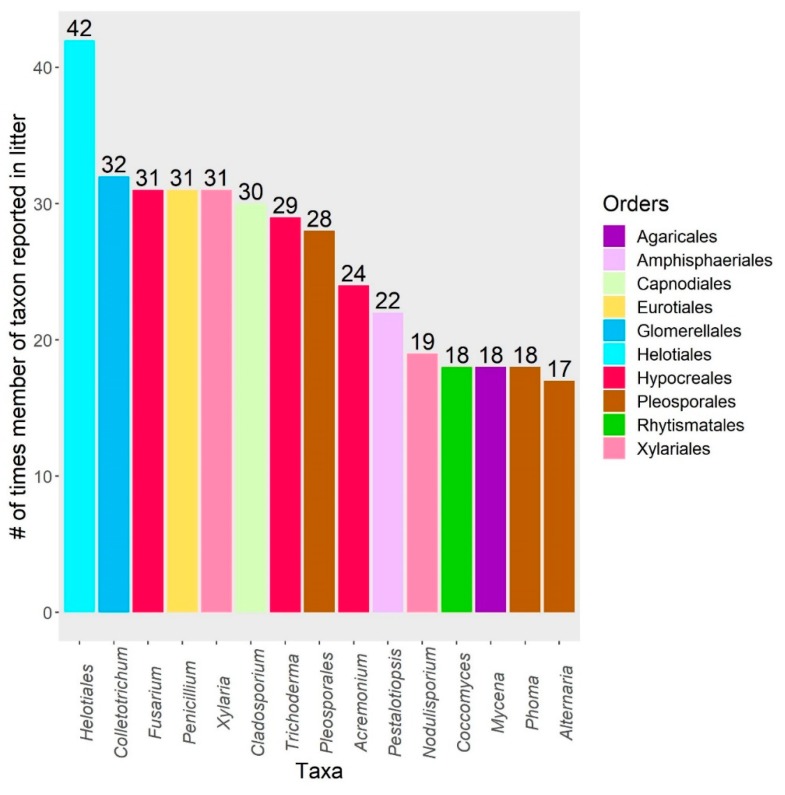
The 15 most frequently reported taxa from leaf litter (e.g., senescent tissue, dead leaves, dried leaves, fresh litter, and decaying litter). Bars represent the number of times members of a taxon were reported. Taxa identified as “undetermined” or “Fungal sp.” are excluded.

**Figure 3:**
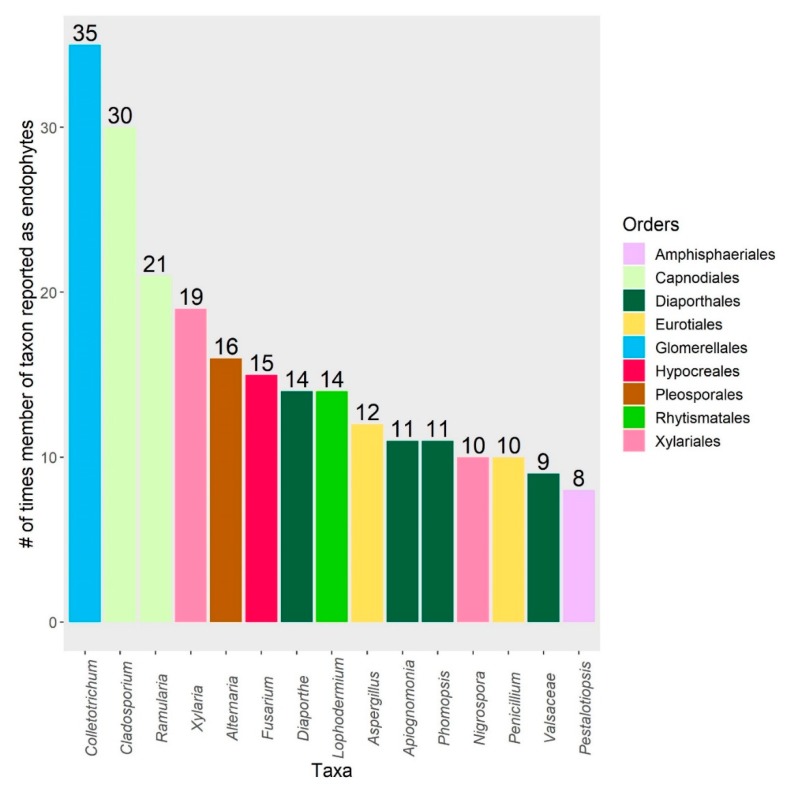
These are the 15 taxa most frequently reported as endophytic (leaves were surface-sterilized). Bars represent the number of times members of a taxon were reported. Taxa identified as “undetermined” or “Fungal sp.” are excluded.

In February 2019, we returned 77 results after searching the following keywords in Web of Science: “endophyte”, “litter”, and “decomposition”. Of the 77 total results, 10 studies were excluded due to irrelevancy, as they did not report foliar microbial community composition or decomposition rates. The remaining 67 studies—spanning 25 years—were subsequently included in this review. The search results are reported in Table S1, and span from 1994 to 2019. A table of 710 leaf-associated taxa was compiled from 29 studies and 1 review (Table S2; raw data available in Table S3) and expands upon Table 2 presented by Osono [18]. “Taxa” refers to the particular taxonomic level identified by the original study authors; to simplify reporting, only classifications at the genus-level or above are included in figures. Reported taxonomic names of species were cross-referenced in MycoBank (export date: 31 July 2019), and updated for improved consistency. The fifteen taxa most frequently reported as having decomposition abilities from litter and as endophytes are shown in Figure 1, Figure 2 and Figure 3, respectively. We chose not to use meta-analysis techniques due to the small sample size of relevant experimental studies (*n* = 14), which would have been further reduced by inconsistent reports of statistical data that would have limited the ability to calculate effect sizes. Finally, we classified the ecosystem climate type for each of the 30 publications mined for taxa, grouping studies into three broad categories: cool climate (e.g., boreal), temperate climate, and tropical climate. We conducted indicator species analyses (R v. 3.6.1, indicspecies package) for these climate types, as well as for grouping by host genera.

## 3. Results

Our search yielded studies of endophytes in diverse climates, forest types, and in both grass and non-grass hosts, with varying effects on litter decomposition rates. Several studies (and one review) provided either brief overviews of endophyte effects on litter decomposition [19,20,21] or general contributions of endophytes to changes in soil microbial communities [22], particulate organic matter [23], and soil organic carbon [24,25], rather than directly referring to litter decomposition. Of the studies that reported taxa, 14 were conducted in temperate forests [26,27,28,29,30,31,32,33,34,35,36,37,38,39], 7 in tropical or subtropical forests [40,41,42,43,44,45,46], and 3 in boreal or subboreal/subalpine forests [47,48,49], with the remaining studies conducted in various forest types (maritime–continental, old-growth, oak, and mountainous forests, respectively) in Europe [50,51,52,53] and a *Cinnamomum* plantation in China [54]. Of these, 13 studies focused primarily on litter microbial community, while 14 studies incorporated some measure of litter decomposition rates. However, out of 67 relevant results, only 14 studies directly tested decomposition of endophyte-affected litter, with about half reporting increased rates [28,55,56,57,58,59,60,61] and the other half decreased rates [15,29,38,49,62,63]. The numbers of studies that reported either increased rates or decreased rates were not statistically significant (binomial exact test, *p* > 0.05). Mikola et al. [64] reported no effects on decomposition rate, even after swapping endophyte-infected litter into endophyte-free plots. Finally, three studies by LeRoy et al. [29], Grimmett et al. [38], and Wolfe et al. [28] were the only in-stream (aquatic) studies that were statistically significant (binomial exact test, *p* < 0.0001). Only one study [28] reported the bacterial community composition, which was also statistically significant (binomial exact test, *p* < 0.0001).

From the 30 publications that we mined, 710 taxa were reported from 25 different host species spanning cool, temperate, and tropical climates (Tables S2 and S3). We found that reports of *Peniophora* and *Zalerion* were indicative of studies conducted in cool forests (e.g., boreal or subalpine), while reports of *Fusarium*, *Phomopsis*, *Idriella*, *Dactylaria*, *Acremonium*, *Cryptophiale*, *Thozetella*, *Mycoleptodiscus*, *Volutella*, and *Verticillium* were specific to studies conducted in tropical forests (indicator species analysis, *p* < 0.05). Several taxa were significantly associated with particular host genera (Table 1). Overarching trends identified in the search results include endophyte effects on litter chemistry, interactions with detritivores and microbial decomposers, and fungal succession patterns in litter, which are addressed below.

## 4. Discussion

### 4.1. Endophytes and Litter Chemistry

Litter chemistry directly influences decomposition rates by altering interactions with detritivores and microbes [9]. Many Class 1 systemic endophytes produce toxic alkaloids in grass hosts, which are thought to contribute to slower decomposition rates. However, while alkaloid concentrations in live tissues are a major concern for grazing livestock, concentrations may decrease following senescence [65] and, therefore, may not directly affect decomposition. It was suggested, however, that N-rich alkaloids could act as a nutrient pulse and stimulate decomposer communities [16]. Conversely, changes in C:N ratios and phenolics—including those induced by plant defenses against microbial pathogens—do persist in litter with well-studied, recalcitrant effects when endophyte status is not considered [66,67]. High C:N ratios typically result in slower decomposition, but in Class 1 endophyte-infected (E+) *Schedonorus pratensis* litter, Gundel et al. [58] reported higher C:N ratios and faster overall decomposition rates compared to E- (endophyte-free) litter, suggesting that other factors may influence endophyte-mediated effects in host grasses. Gundel et al. [58] also measured lower N concentrations in E+ litter, but Soto-Barajas et al. [68] found that symptomatic endophyte infection (i.e., endophytes that have transitioned to a symptomatic infection of host plant tissues; [69]) increased N concentrations in *Lolium perenne*, indicating that the type of and structures associated with infection are important considerations in predicting decomposition effects. Similar results were reported by LeRoy et al. [29] for *Rhytisma punctatum*-infected punches of bigleaf maple (*Acer macrophyllum*) litter, in which punches with symptomatic Class 3 infections had significantly higher N content, but significantly lower C:N ratios compared to nearby or uninfected patches. Soto-Barajas et al. [68] did not measure decomposition rates, but LeRoy et al. [29] found that symptomatic Class 3 infection retarded in-stream decomposition. Additionally, phenolic compounds are produced as defenses against microbial pathogens; high concentrations in litter also typically slow decomposition. In poplar (*Populus* sp.) and Norway spruce (*Picea abies*) trees, increased concentrations of phenolics contributed to decreased foliar endophyte presence [50,70]. Bailey et al. [70] specifically reported differences in leaf chemistry among different genotypes of poplar (see [71]), while Korkama-Rajala et al. [49] found that clone origin affected fungal community composition. Finally, while widely recognized for vertically transmitted Class 1 endophytes, coevolutionary relationships between host taxa and specific Class 2 and 3 endophytes may further influence community composition, as reviewed by Sieber [72] and supported here by the results of our indicator species analyses in Table 1. Consequently, plant host genotypes, origin, and host–endophyte coevolutionary relationships may be considered alongside endophyte community composition to interpret patterns in litter decomposition rates.

### 4.2. Endophytes as Decomposers (Interactions with Detritivores and Microbial Decomposers)

Seven of the studies from the search results specifically investigated the ability of isolated Class 2 and 3 endophytes to decompose leaf litter ([28,29,38,56,57,60]; Figure 1). While most species of endophytes may not persist into the later stages of decomposition, some endophytes are capable of directly participating in the process, in competition with persisting epiphytes and new colonizers [73]. Importantly, all of the isolated strains used in the studies had some effect on litter decomposition and produced several extracellular enzymes, including cellulases, laccases, and β-glucosidases [43,51,57,74]. *Colletotrichum* sp. [57] and *Coccomyces* sp. [33] were dominant strains that stimulated litter decomposition when inoculated alone or as the initial colonizer, respectively. Sun et al. [60] also found *Phomopsis liquidambari* to be capable of increasing decomposition of straw from rice plants, but only under conditions of low to moderate nitrogen. Additionally, Chen et al. [56] found that the same endophyte increased the concentrations of phenolics in the soil, subsequently affecting the composition of the microbial decomposer community by reducing soil fungi and increasing bacteria during the early stages of decomposition. This shows that in addition to competing directly, persisting endophytes can also have allelopathic effects on other microbes, although the specific effects of phenolic compounds on microbes is context-dependent, in that it matters which compounds and microbes are present [75,76].

Two in-stream studies also found negative effects of endophytes on litter decomposition, with one study specifically focusing on the fungal decomposer community [29,38]. Aquatic hyphomycetes are the primary in-stream decomposers of leaf litter, and their sporulation rates were significantly reduced by symptomatic Class 3 endophyte infection by *Rhytisma* sp. in *Acer* sp. litter [28,38]. However, Wolfe et al. [28] reported faster rates of decomposition for litter with symptomatic Class 3 infections and suggested macroinvertebrate presence as a contributing factor, given that Lemons et al. [15] had previously reported negative effects in Class 1 E+ *Lolium arundinaceum* litter in the absence of mesodetritivores. Detritivores also appear to play an important role in mediating at least Class 1 endophyte-produced secondary compounds; Mayer at al. [55] found that macrodetritivore abundance increased with the presence of *L. arundinaceum* in plots and contributed to increased decomposition rates of herbaceous litter. Increased arthropod abundance was also supported by Faeth and Shochat [77] in *Neotyphodium*-infected *Festuca arizonica*. Jackrel and Woontton [78] emphasized that changes in litter chemistry due to plant defense responses can decrease palatability for detritivores, although the effects on decomposition rates differed for aquatic and terrestrial groups.

### 4.3. Endophytes in Litter Decomposer Assemblages

Taxa identified as endophytes in living to decaying leaves are included in Tables S2 and S3 and summarize the results reported in the 29 studies and 1 review returned by our search (Figure 2 and Figure 3). The presence of endophytes and epiphytes was previously reported in leaf litter from various species, and fungal succession on decomposing leaves was reviewed by Osono [18]. However, the bacterial phyllosphere community during in-stream decomposition was only reported by one study in our search [28], which presents an opportunity for future work. Osono and Takeda [26] and Osono [27] identified xylariaceous endophytes as significant contributors to terrestrial litter decomposition due to their abilities to persist from living tissues and decompose lignin. Reviews by Purahong and Hyde [79] and Saikkonen et al. [80] also recognized endophytes as potential saprophytes, which is a role governed by specific nutrient requirements and the capacity to produce certain classes of extracellular enzymes. Additionally, some endophyte species are considered to be latent pathogens and can switch lifestyles based on the presence of environmental stressors [81]. Hagiwara et al. [48] and Matsukura et al. [82] each surveyed ligninolytic endophytes that caused bleaching in up to 32% of measured leaf area. Switches to symptomatic endophyte infections are important considerations, since they were shown to affect decomposition rates differently than asymptomatic tissue [28,29,68]. Chauvet et al. [83] and Seena and Monroy [84] also reviewed the occurrence of aquatic hyphomycetes living as endophytes. This occurrence has interesting implications for in-stream decomposition, since it is largely mediated by aquatic hyphomycetes in the initial stages. However, Mustonen et al. [47] found that, while aquatic hyphomycete taxa were present, terrestrial endophytes dominated the sequenced litter decomposer communities under low-flow conditions and may have contributed to the greater mass loss observed. Tateno et al. [35] also isolated similar endophytes from twigs, leaves, and cupules in beech, suggesting that horizontally transmitted foliar endophytes communities are likely influenced by propagules in other plant parts. Importantly, Guerreiro et al. [30] found that endophyte communities are linked to and influenced by fungal communities in litter; endophytic fungi were still present and active in one-year-old litter.

## 5. Conclusions

Do foliar endophytes matter in litter decomposition? Our review of the current literature suggests that it depends, given the complexity of abiotic and biotic factors influencing ecosystem processes. While it is important to point out that there were only 12 studies that directly tested foliar endophyte effects on litter decomposition—half of which focused exclusively on Class 1 grass–endophyte interactions—our synthesis suggests that there is an overarching theme of mismatched focus among studies of litter decomposition. Endophytes are a hyperdiverse group of organisms that includes both bacteria and fungi, colonizing a wide range of host plants and plant tissues from tropical to boreal ecosystems. These microbes exist within the phyllosphere of their host plants and emphasize just one example from the tangled web of plant–microbe interactions. A host plant can represent a patch of occupiable habitat to an endophytic colonizer. Within that patch, there is competition with other endophytes and parasites, but also specific host–endophyte interactions. These specific interactions are, in turn, complicated by variation in the endophyte community composition and the host responses to abiotic factors. However, the resolution of the “patch” unit matters. For example, endophytes can vary spatially within the same host plant, shifting the occupiable patch unit to leaf. Similarly, endophytes within the local litter community can colonize neighboring host plants, shifting the occupiable patch unit to a localized area. Because endophyte–host interactions span more than a single level within the scale of an ecosystem—and ultimately represent just one of many poorly defined mechanisms and interactions between different scales within an ecosystem—endophyte communities present special challenges to predicting and understanding ecosystem processes like litter decomposition.

**Figure 4:**
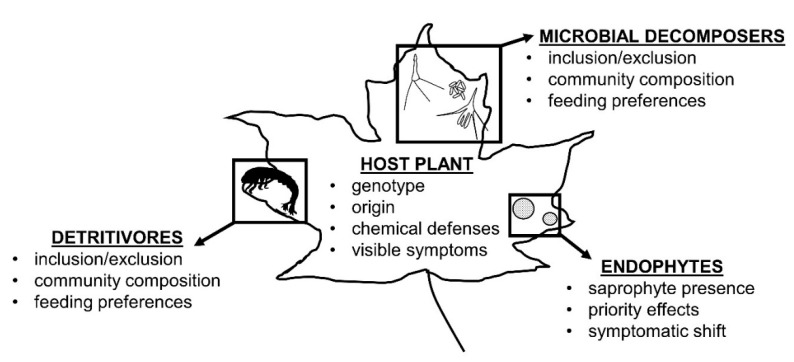
Infographic of factors that influence endophyte-mediated litter decomposition.

Several key contributing factors should be considered in litter decomposition studies when designing experiments or interpreting results (Figure 4). First, host–endophyte interactions are context-dependent, and may be influenced by the host’s genotype or origin and both abiotic and biotic factors that affect leaf chemistry (e.g., drought or herbivores). The presence of secondary compounds—whether produced as host defenses or induced by systemic Class 1 endophyte infection (in grasses)—tend to slow litter decomposition overall in terrestrial habitats. Similarly, symptomatic endophyte infections in litter typically slow decomposition dynamics by inhibiting subsequent colonization or directly breaking down recalcitrant compounds. However, saprotrophic endophytes can both exert priority effects on new colonizers and ameliorate available nutrients on litter; as common members of the foliar endophyte communities, their presence should be considered, especially in studies of microbial decomposer succession or community assembly on litter. Finally, access by detritivores is important in mediating litter decomposition rates, particularly when unpalatable or recalcitrant compounds are present and would otherwise retard microbial decomposition.

Most importantly, affecting all of these contributing factors is the type of ecosystem itself (e.g., riparian versus terrestrial, or grassland versus forest). Decomposition proceeds faster in aquatic environments due to a combination of factors including constant moisture and mechanical breakdown from moving water. Likewise, warm ambient temperatures and high humidity tend to accelerate decomposition in tropical forests. In grass systems, other factors, such as variable precipitation regimes and agricultural land use, must be considered, especially since the presence of toxic alkaloids can harm grazing livestock. Grasses also harbor Class 1 endophytes, which are vertically transmitted, as opposed to the horizontal transmission of Class 2 and 3 endophytes that are more prevalent in non-grasses. We found that climate type—even broadly categorized—resulted in significantly different groups of specialized taxa. Different plant genera also tended to host specialist fungal taxa, in addition to many well-known generalist endophytes (e.g., *Phoma* and *Xylaria*). In summary, host plant and endophyte interactions can be significant factors in both terrestrial and aquatic litter decomposition rates and should be taken into consideration when interpreting results, but more studies specifically exploring foliar endophyte effects on litter decomposition are clearly needed. 

## Figures and Tables

**Table 1:** Taxa significantly associated with host genera.

Taxa	Associated Host Genus	Fungal Order	Indicator Value	*p*-Value
*Rhytisma*	*Acer*	Rhytismatales	0.816497	0.005
*Boeremia*	*Alnus*	Pleosporales	0.912871	0.005
*Ophiognomonia*	*Alnus*	Diaporthales	0.5	0.015
*Pseudopithomyces*	*Alnus*	Pleosporales	0.5	0.03
Amphisphaeriaceae	*Fagus*	Xylariales	0.57735	0.025
*Apiognomonia*	*Fagus*	Diaporthales	0.57735	0.025
*Arthrinium*	*Fagus*	Sordariales	0.5	0.015
*Ascochyta*	*Fagus*	Pleosporales	0.745356	0.005
*Beauveria*	*Fagus*	Hypocreales	0.57735	0.025
*Cryptococcus*	*Fagus*	Tremellales	0.537484	0.045
*Cylindrium*	*Fagus*	Hypocreales	0.447214	0.05
*Discosia*	*Fagus*	Amphisphaeriales	0.547723	0.01
Dothideomycetes	*Fagus*	NA	0.471405	0.05
Fungal sp. (undetermined)	*Fagus*	NA	0.573026	0.025
*Geniculosporium*	*Fagus*	Xylariales	0.645497	0.005
*Hypoxylon*	*Fagus*	Xylariales	0.456436	0.035
*Mycosphaerella*	*Fagus*	Capnodiales	0.707107	0.005
Nectriaceae	*Fagus*	Hypocreales	0.544331	0.025
Pezizomycotina	*Fagus*	NA	0.57735	0.025
*Phaeosphaeria*	*Fagus*	Pleosporales	0.447214	0.04
*Phialemoniopsis*	*Fagus*	Cephalothecales	0.57735	0.025
*Hormonema*	*Picea*	Dothideales	0.632456	0.005
*Lachnum*	*Picea*	Helotiales	0.489898	0.03
*Lophodermium*	*Picea*	Rhytismatales	0.6	0.005
*Rhizoctonia*	*Picea*	Cantharellales	0.516398	0.02
*Thysanophora*	*Picea*	Eurotiales	0.632456	0.005
*Rhabdocline*	*Pseudotsuga*	Helotiales	0.894427	0.005
*Helminthosporium*	*Quercus*	Pleosporales	0.516398	0.02

## Supplementary Materials

The following are available online at https://www.mdpi.com/2076-2607/8/3/446/s1, Table S1: Exported citation report generated from Web of Science for the keyword search: endophyte litter decomposition, Table S2: Leaf-associated taxa compiled from 29 studies and 1 review, and Table S3: Raw data collection sheet.
